# Consistency of aortic distensibility and pulse wave velocity estimates with respect to the Bramwell-Hill theoretical model: a cardiovascular magnetic resonance study

**DOI:** 10.1186/1532-429X-13-11

**Published:** 2011-01-27

**Authors:** Anas Dogui, Nadjia Kachenoura, Frédérique Frouin, Muriel Lefort, Alain De Cesare, Elie Mousseaux, Alain Herment

**Affiliations:** 1 Inserm U678, 91 boulevard de l'Hôpital, 75634 Paris cedex 13, France; 2 AP-HP, Service de Radiologie Cardiovasculaire, Hôpital Européen Georges Pompidou, 20 rue Leblanc, Paris, 75015, France

## Abstract

**Background:**

Arterial stiffness is considered as an independent predictor of cardiovascular mortality, and is increasingly used in clinical practice. This study aimed at evaluating the consistency of the automated estimation of regional and local aortic stiffness indices from cardiovascular magnetic resonance (CMR) data.

**Results:**

Forty-six healthy subjects underwent carotid-femoral pulse wave velocity measurements (*CF_PWV*) by applanation tonometry and CMR with steady-state free-precession and phase contrast acquisitions at the level of the aortic arch. These data were used for the automated evaluation of the aortic arch pulse wave velocity (*Arch_PWV*), and the ascending aorta distensibility (*AA_Distc, AA_Distb)*, which were estimated from ascending aorta strain (*AA_Strain*) combined with either carotid or brachial pulse pressure. The local ascending aorta pulse wave velocity *AA_PWVc *and *AA_PWVb *were estimated respectively from these carotid and brachial derived distensibility indices according to the Bramwell-Hill theoretical model, and were compared with the *Arch_PWV*. In addition, a reproducibility analysis of *AA_PWV *measurement and its comparison with the standard *CF_PWV *was performed. Characterization according to the Bramwell-Hill equation resulted in good correlations between *Arch_PWV *and both local distensibility indices *AA_Distc *(r = 0.71, p < 0.001) and *AA_Distb *(r = 0.60, p < 0.001); and between *Arch_PWV *and both theoretical local indices *AA_PWVc *(r = 0.78, p < 0.001) and *AA_PWVb *(r = 0.78, p < 0.001). Furthermore, the *Arch_PWV *was well related to *CF_PWV *(r = 0.69, p < 0.001) and its estimation was highly reproducible (inter-operator variability: 7.1%).

**Conclusions:**

The present work confirmed the consistency and robustness of the regional index *Arch_PWV *and the local indices *AA_Distc and AA_Distb *according to the theoretical model, as well as to the well established measurement of *CF_PWV*, demonstrating the relevance of the regional and local CMR indices.

## Background

Changes in aortic stiffness have a high physiopathological relevance as they can lead to increases in the aortic pulse pressure [1,2] and the cardiac pressure afterload, which can cause left ventricular hypertrophy [3]. Arterial stiffness is recognized as a major risk factor in coronary heart disease [4,5], and is considered as an independent predictor of cardiovascular mortality [6-10]. It is therefore increasingly used in clinical practice [11]. Distensibility and pulse wave velocity (PWV) are commonly used to characterize the arterial stiffness [12-16]. The distensibility describes the ability of the artery to expand during systole, and is defined as the relative change in the cross-sectional area of the artery (strain) divided by the local pulse pressure. The PWV is the propagation speed of the pressure or the velocity wave along the artery, and is calculated as the ratio between the distance separating two locations and the transit time needed for the wave to cover this distance.

Tonometry is the most commonly used technique for quantification of global vascular function [11]. However, this technique can only provide a global estimation of the aortic PWV, along the whole carotid-femoral artery path. Indeed, tonometry uses body surface anatomy to estimate artery length and does not take into account the often torturous route of the vessels.

Cardiovascular magnetic resonance (CMR) is increasingly used to analyze the local and regional mechanical properties of the aortic wall and the blood flow [13-24]. Steady-state free-precession (SSFP) cine acquisitions enable a direct estimation of the aortic strain at localized and specific levels of the thoracic aorta, as well as the precise measurement of the length of the aorta. Furthermore, phase-contrast (PC) cine acquisitions provide an accurate assessment of the blood flow velocities throughout different aortic sections during the cardiac cycle, which enable the estimation of velocity waveforms. The transit time of a velocity waveform propagation between two aortic sections can be calculated and its combination with the aortic distance travelled by the waveform provides the aortic arch PWV[15]. The combination of the aortic strain with pulse pressure measurements results in local aortic distensibility.

Although the relation between the aortic strain and the distending pressure is complex because the aorta may exhibit a non-linear and spatially non-uniform elastic behavior, a theoretical model that links the PWV, strain, pulse pressure, and blood density have been proposed by Bramwell and Hill [25] and have been commonly used in clinical practice [11]. Despite the fact that the Bramwell and Hill equation was derived from the Moens-Korteweg equation [26], more modern theoretical work using the 1-D equations describing flow in compliant vessels [26] shows that the Bramwell-Hill model is more general since it does not consider assumptions such as thin-walled and homogeneous elastic arteries that are assumed in the Moens-Korteweg model.

Accordingly, our primary goal was to use the theoretical model described by Bramwell and Hill [25] to demonstrate the consistency of the automated MR measurements of the local and regional aortic stiffness indices. Furthermore, comparisons against the clinical standard of the tonometric carotid-femoral PWV (*CF_PWV*) estimates as well as a reproducibility analysis of the MR PWV measurements were performed.

## Methods

### Data acquisition

For this study, 46 volunteers (age: 39 ± 15 years) were recruited. None of the volunteers had any history of cardiovascular events or hypertension. All CMR examinations were performed on a 1.5T scanner (Sigma LX; General Electric Medical Systems, Milwaukee, Wisconsin, US) using a cardiac phased-array coil and ECG-gated sequences. SSFP and PC cine acquisitions were acquired for each subject.

For the local ascending aortic strain (*AA_Strain*) measurements, an axial dataset was positioned perpendicular to the axis of the aorta at the level of the bifurcation of the pulmonary trunk (between 2 and 4 cm above the aortic junction) to ensure optimal imaging quality [27] and to avoid distortion due to the aortic valve movement. Axial dataset was acquired according to the SSFP sequence using the following average scan parameters: field-of-view = 370 mm × 370 mm, repetition time = 3.2 ms, echo time = 1.4 ms, flip angle = 50°, slice thickness = 8 mm, pixel size = 1.65 mm × 1.92 mm, and inter phase duration = 33 ms. For further evaluation of the aortic geometry, axial and coronal sequences covering the whole aortic arch were acquired using the same protocol. For the aortic arch *PWV *(*Arch_PWV*) estimation, the PC slice was set at the level used for ascending aortic strain measurement. Hence, the ascending and descending aorta could be studied simultaneously. The PC data were acquired using a retrospectively ECG-gated breath-hold gradient sequence with a velocity encoding gradient in the through-plane direction, which provided phase-related pairs of modulus and velocity-encoded images. The scan parameters were: repetition time = 9 ms, echo time = 3.5 ms, flip angle = 20°, views per segment = 2, rectangular field-of-view = 50%, acquisition matrix = 256 × 128, pixel size = 1.58 mm × 1.58 mm, slice thickness = 8 mm, and encoding velocity = 200 cm/s. View sharing was used resulting in an effective temporal resolution of 18 ms.

Oscillometric techniques were used to assess the pulse pressure at the brachial artery (Vital Signs Monitor, Welch Allyn Inc, US). The brachial pressure was measured with a sensor cuff in the magnet simultaneously to MR SSFP and PC acquisitions of the aorta.

Applanation tonometry of both the right carotid artery and right femoral artery was performed with the Pulse Pen device (Diatecne, Milano, Italy) [28] immediately after the MRI acquisitions. These acquisitions were used to estimate the carotid pulse pressure, and the carotid-femoral PWV (*CF_PWV*). The *CF_PWV *was defined as the ratio of the difference between the suprasternal notch-femoral and the carotid-suprasternal notch distances, to the transit time between the pressures waveforms recorded at the carotid and femoral arteries. The suprasternal notch-femoral, and the carotid-suprasternal notch distances were measured by means of a tape ruler over the body surface [29]. The transit time was measured as the foot-to-foot interval of the carotid and femoral waveforms. The carotid pulse pressure was calculated after rescaling tonometric measurement by the brachial mean and diastolic pressures measured simultaneously to the MR acquisitions. This rescaling is based on the assumption that the difference between the mean and diastolic blood pressures remains unchanged throughout the arterial artery pathway [11].

### Theoretical model

Bramwell and Hill [25] proposed an equation which links the pulse wave velocity with the vessel and fluid characteristics. It assumes that the vessel is compliant and filled with an incompressible nonviscous fluid.

(1) $$PWV=\frac{1}{\sqrt{\rho \times Distensibility}}.$$

Here *ρ *is the blood density (1059 kg.m^-3^). The Distensibility of an infinitesimally thin slice of the vessel under the pressure of a fluid is computed as follow:

(2) $$Distensibility=\frac{strain}{\Delta P}=\frac{\Delta S}{S\times \Delta P}.$$

Here, Δ*P *is the pulse pressure, and strain is the induced relative variation of the vessel cross-sectional area (Δ*S/S*).

The Bramwell-Hill equation (1) was derived from the Moens-Korteweg equation [26], and is commonly used in clinical studies [11].

In our study, the equation (1) was applied on the ascending aorta to study the relationship between the PWV along the aortic arch (*Arch_PWV*) and the direct measurement of the local ascending aortic strain (*AA_Strain*), combined with either brachial or carotid pulse pressure.

### CMR image analysis

Local and regional indices of aortic stiffness were estimated from MR SSFP and PC cine images using the following post-processing approaches.

### Regional aortic arch pulse wave velocity

To extract ascending and descending aorta mean velocity curves, aortic lumen contours were detected from the modulus of the PC images using the Art-FUN software package as described in a previous work [30]. Contours were then superimposed on the velocity-encoded images.

The aortic arch PWV (*Arch_PWV*) was calculated as the ratio between the 3D length of the aortic arch, and the transit time (Δt) between the velocity waveforms in the ascending and descending aorta.

**Figure 1:**
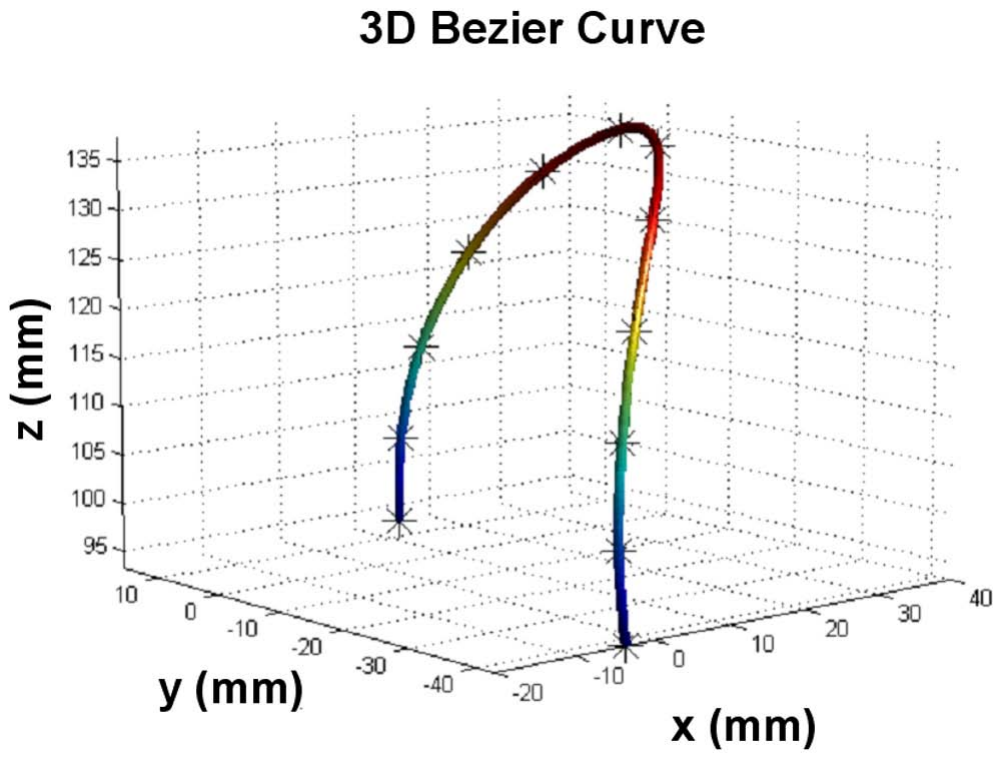
**Estimation of the aortic length**. Estimation of the aortic length from the axial and coronal slices with a 3D Bezier curve interpolation

To estimate the 3D length of the aortic arch, the centers of the aortic lumen were first selected by an experienced user on each SSFP axial and coronal slices in a 3D Coordinate-System. Six to eight markers were defined for the ascending and the descending segment on axial slices, and three markers were defined for the top of the aortic arch on coronal slices. The 3D coordinates of the selected centers were computed from the DICOM headers of the MR images, and were interpolated with a 3D Bezier curve (Figure 1). The length of the 3D Bezier curve comprised between the ascending and descending aorta planes defined from the PC images was considered for the estimation of the *AA_PWV*.

As previously described [31], to minimize the variability of foot-to-foot measurement inherent to lower temporal resolution on mean velocity curves compared to pressure curves, the transit time (Δ*t*) was calculated automatically using a method based on the least squares minimization approach between the systolic up-slope of the ascending aorta mean velocity curve, and the whole descending aorta mean velocity curve. The systolic up-slope was defined as the portion of the mean velocity curve comprised between the onset of the blood flow and the time of its maximum. This up-slope portion was preferred to the entire flow curve because of the unidirectional and reflectionless nature of the flow wave during this systolic phase as shown in [16,26].

First, the mean velocity waveforms were re-sampled to a temporal resolution of 1 ms using a cubic interpolation, and were normalized to account for the differences between the waveform amplitudes. Then, the transit time (Δ*t*) was calculated as the time shift for which the resemblance between the profile of the systolic up-slope of the normalized mean ascending aorta velocity waveforms ($E_{A}^{*}$), and the normalized descending aorta waveform (*E_D_*) was maximal. This maximal resemblance was obtained by shifting *E_D _*by successive temporal translations with a unitary step of 1 ms, and by minimizing the quadratic error (*Er*) between $E_{A}^{*}$ and *E_D_*.

(3) $$Er(k)=\frac{1}{N}\sum_{i=1}^{N}{\left(E_{A}^{*}(i)-E_{D}(i+k)\right)}^{2}.$$

Here, *k *is the temporal translation, and *N *is the number of samples of the systolic up-slope $E_{A}^{*}$.

The transit time Δ*t *was then provided as follows:

(4) $$\Delta t=k^{+}\;with\;Er(k^{+})=\underset{k\in [0,100]}{\min}\left\{Er(k)\right\}.$$

The estimation of the aortic arch length and the transit time was repeated by two independent operators to assess inter-observers variability.

### Local ascending aorta strain and distensibility

**Figure 2:**
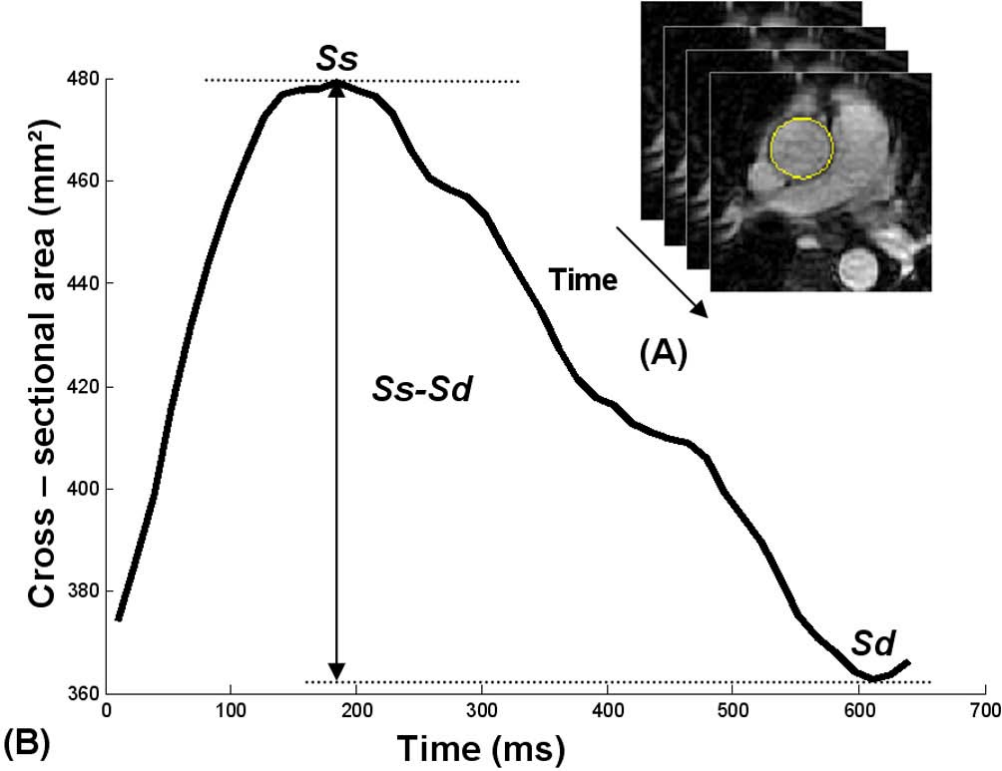
**Determination of the aortic cross-sectional area during a cardiac cycle**. (A): automatic contouring of the ascending aorta. (B): ascending aorta cross-sectional area versus time curve; *Ss *and *Sd *correspond to the ascending aorta systolic and diastolic areas, respectively.

The local aortic strain (*AA_Strain*) was calculated from the systolic (*Ss*) and diastolic (*Sd*) areas of the aortic lumen at the level of the ascending aorta (*AA_Strain = (Ss-Sd)/Sd). *These lumen areas were measured from SSFP cine MR acquisitions using the automatic segmentation previously used on PC modulus images [30]. The *Ss *and *Sd *areas were defined as the maximum and the minimum of the curve describing the lumen area variation during the cardiac cycle (Figure 2).

According to the equation (2), the combination of the resulting strain with either the tonometric carotid pulse pressure or the brachial pulse pressure provided the ascending aorta distensibility indices, respectively named *AA_Distc *and *AA_Distb*.

### Local theoretical ascending aorta pulse wave velocity

The local theoretical indices of ascending aorta PWV *AA_PWVc *and *AA_PWVb *were estimated according to the Bramwell-Hill equation (1) from the above local indices of ascending aorta distensibility.

### Statistical analysis

Comparisons were performed using linear regression analysis, as well as mean ± standard deviation (SD). For regression analysis Pearson's correlation coefficient^® ^was provided, and statistical significance was indicated by p < 0.05. The inter-observer variability was studied using the coefficient of variation defined as the standard deviation of the differences between two series of measurements divided by the mean of the measurements.

## Results

The characteristics as well as the estimated aortic and arterial stiffness indices obtained for the 46 volunteers are summarized in Table 1.

**Table 1:** Subjects characteristics

Parameters	Subjects (n = 46)
Age (years)	39 ± 15
Body mass index (kg.m^-2^)	23.75 ± 3.4
Aortic length (cm)	12.1 ± 2.1
Carotid pulse pressure (mmHg)	35.9 ± 10.6
Brachial pulse pressure (mmHg)	45.4 ± 10
*Arch_PWV *(m.s^-1^)	4.34 ± 1.29
*CF_PWV *(m.s^-1^)	7.07 ± 3.19
*AA_Distc *(10^-3 ^mmHg^-1^)	5.86 ± 3.23
*AA_Distb *(10^-3 ^mmHg^-1^)	4.52 ± 2.4
*AA_PWVc *(m.s^-1^)	5.55 ± 2.55
*AA_PWVb *(m.s^-1^)	6.26 ± 2.83

Subjects characteristics are expressed as Mean ± SD.; *Arch_PWV*: regional aortic arch PWV assessed with MRI; *CF_PWV*: global aortic PWV assessed with tonometry; *AA_Distc *and *AA_Distb*: local ascending aorta distensibility estimated from MRI aortic strain and carotid or brachial pulse pressures; *AA_PWVc *and *AA_PWVb*: local ascending aorta PWV estimated according equation (1) from *AA_Distc *or *AA_Distb*.

### *Arch_PWV *estimation

Reproducibility of the CMR regional *Arch_PWV *index was measured by repeating the estimation of the aortic length and the transit time by two operators. The average length of the aortic arch was 12.1 ± 2.1 cm for the first operator, and 12.3 ± 2.1 cm for the second operator; providing a coefficient of variation of 4.0%. The average transit time was 29 ± 5 ms for the first operator, and 28 ± 5 ms for the second operator; providing a coefficient of variation of 4.4%. These two parameters resulted in a coefficient of variation of 7.1% for the estimation of the *Arch_PWV*. Furthermore, the linear regression analysis between this latter parameter and the tonometric *CF_PWV *resulted in a correlation of r = 0.69 (p < 0.001). The equation of the linear regression was *Arch_PWV *= 0.28× *CF_PWV *+2.33.

### Comparison between regional index *Arch_PWV *and both local indices *AA_Distc *and *AA_Distb*

**Figure 3:**
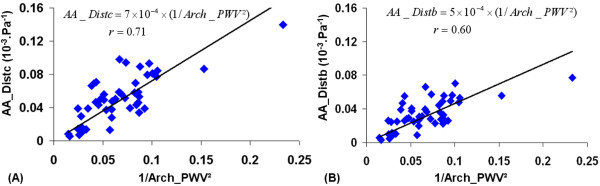
**Correlations between local ascending aorta distensibility and regional aortic arch PWV**. (A): comparison between *AA_Distc *and *1/Arch_PWV^2^*. (B): comparison between *AA_Distb *and *1/Arch_PWV^2^*. *AA_Distc *and *AA_Distb*: local ascending aorta distensibility estimated from MRI aortic strain and carotid or brachial pulse pressures; *Arch_PWV*: regional aortic arch PWV assessed with MRI.

Given the Bramwell-Hill equation (1), linear regressions between local ascending aortic distensibility indices *AA_Distc*, *AA_Distb*, respectively, and *1/Arch_PWV^2 ^*were performed and shown in figure 3. The relationship between these indices resulted in a Pearson coefficient of r = 0.71 (p < 0.001) between *AA_Distc *and *1/Arch_PWV^2 ^*; and of r = 0.60 (p < 0.001) between *AA_Distb *and *1/Arch_PWV^2^*. In addition, when the carotid pulse pressure was used for the estimation of the local ascending aorta distensibility, the slope (7×10^-4^) of the linear regression was closer to theoretical value calculated from the blood density in Bramwell-Hill equation (1) (1/ρ = 9×10^-4^). This slope was equal to 5×10^-4 ^when the brachial pulse pressure was used for the estimation of the local ascending aorta distensibility.

### Comparison between regional index *Arch_PWV *and both local indices *AA_PWVc *and *AA_PWVb*

**Figure 4:**
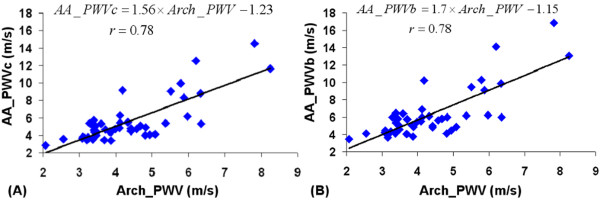
**Correlations between local ascending aorta PWV and regional aortic arch PWV**. (A): comparison between *AA_PWVtc *and *Arch_PWV*. (B): comparison between *AA_PWVb *and *Arch_PWV*. *AA_PWVc *and *AA_PWVb*: local ascending aorta PWV estimated according equation (1) from *AA_Distc *or *AA_Distb*; *Arch_PWV*: regional aortic arch PWV assessed with CMR.

The local aortic pulse wave velocity *AA_PWVc *and *AA_PWVb *indices were compared with the regional *Arch_PWV *index, resulting in Pearson's correlation coefficient of r = 0.78 (p < 0.001) for both *AA_PWVc *and *AA_PWVb*. These comparisons are shown in Figure 4. However, the regression analysis indicated that the regional *Arch_PWV *was lower than both the local theoretical *AA_PWVc *and *AA_PWVb*. As shown in Table 2, these differences were more important for the highest values of PWV. Indeed, when considering subject < = 50 years old (n = 37), the mean value of these differences decreases from 1.21 (SD = 1.71) to 0.75 (SD = 1.06) for *AA_PWVc*, and from 1.92 (SD = 1.99) to 1.37 (SD = 1.2) for *AA_PWVb*. Furthermore, the slope of the linear regression was slightly lower when the carotid pulse pressure was used for the estimation of the local ascending aorta PWV.

**Table 2:** Results of the local and regional PWV measurements

Pulse wave velocity	Subjects ≤ 50 years (n = 37)	Subjects >50 years (n = 9)
*Arch_PWV *(m/s)	3.84 ± 0.75	6.39 ± 0.99
*AA_PWVc *(m/s)	4.59 ± 1.11	9.49 ± 3.07
*AA_PWVb *(m/s)	5.22 ± 1.22	10.55 ± 3.57

PWV are expressed as Mean ± SD; Arch_PWV: regional aortic arch PWV assessed with MRI; AA_PWVc and AA_PWVb: local ascending aorta PWV estimated according equation (1) from AA_Distc or AA_Distb.

## Discussion

In this study, the indices of aortic stiffness were assessed automatically and non-invasively using CMR and pulse pressure measurements. The consistency of the CMR indices was investigated using the theoretical model (1), a comparison with the independent measurement of the arterial stiffness using the tonometric *CF_PWV*, and an inter-observer variability analysis.

Although the aortic distensibility (*AA_Distc*, *AA_Distb*) is a local index, while the aortic arch PWV (*Arch_PWV*) is a regional index, their relationships were well characterized according to the Bramwell-Hill model. Indeed, the consistency of our CMR measurements of aortic stiffness was confirmed by the good correlations found when comparing: 1) *1/Arch_PWV ^2 ^*with the local distensibility indices *AA_Distc *and *AA_Distb *estimated from *AA_Strain *and carotid or brachial pulse pressures, and 2) the aortic *Arch_PWV *with the local theoretical indices *AA_PWVc *and *AA_PWVb *estimated according to the equation (1).

Slight differences were found when comparing *AA_PWVc *and *AA_PWVb *with the *Arch_PWV*. These differences which were consistent with results of a previous study that compared *AA_PWVb *against *Arch_PWV*, can be explained by the fact that the comparison was performed between a regional and a local measurement. To reduce these differences, two directional in-plane velocity encoded data [32,33] could be used to calculate the PWV at the same site than the theoretical estimation. In addition, the differences between the local theoretical PWV measurements in the ascending aorta and the regional *Arch_PWV *were more important for subjects with the highest PWV values. This might be due to the more pronounced effect of aging on the ascending aorta [29], where there are more elastin fibers than in the other parts of the aorta [34]. Despite these differences good correlations between the *Arch_PWV *and the local theoretical indices *AA_PWVc *and *AA_PWVb *were found in our study.

Of note, a slope of linear regression closer to (1/ρ) and a higher correlation coefficient were obtained for the comparison between *1/Arch_PWV^2 ^*and *AA_Distc *than for the comparison between *1/Arch_PWV^2 ^*and *AA_Distb*. In addition, lower mean difference and slope closer to 1 were obtained for the comparison between the *Arch_PWV *and the *AA_PWVc *than for the comparison between the *Arch_PWV *and the *AA_PWVb*. These finding indicated that, according to the Bramwell-Hill equation (1) applied on the ascending aorta, the *Arch_PWV *was better related to the ascending aorta distensibility estimated from carotid pulse pressure than from brachial pulse pressure. This might be due to the fact that the carotid pulse pressure assessed by tonometry is more representative for the aortic pulse pressure [11]. Indeed, data from invasive studies showed that carotid artery pulse pressure only slightly overestimate the pulse pressure in the ascending aorta with less than 2 mmHg [35].

In our study, the analysis of the CMR SSFP and PC cine data was performed using a robust segmentation technique based on a (2D+t) snake algorithm. This segmentation technique was previously used for the segmentation of the ascending and descending aorta data that were acquired on three different MR devices, resulting in a very high intra- and inter-observers reproducibility [30]. It has been also validated against manual tracing for both healthy volunteers and patients with dilated aorta [30].

The aortic arch PWV (*Arch_PWV*) was estimated from the length of the aortic arch, and the temporal shift between the mean velocity waveforms in the ascending and descending aorta. In our study, a 3D approach was used to assess the aortic arch length from both the coronal and axial slices, rather than the traditional 2D measurement which is usually performed from a single sagittal oblique section by a manual selection of the centreline of the aorta [13,15-17]. The advantage of our 3D technique is its ability to better take into account the 3D geometry of the aorta. Indeed, the curvature of the aortic arch is not always aligned in a specific plane regarding to the position of the ascending and descending aorta. Since our technique required a manual positioning of the centers of the aorta lumen on axial and coronal data acquired during two successive apneas, its reproducibility was studied, resulting in very low inter-observer variability. The transit time was estimated from the systolic up-slope portion of the velocity waves using an automated method based on a least squares minimization technique used in previous studies for the evaluation of the effect of coarctation repair [20] and aging [21] on CMR aortic stiffness indices. Other methods based on the foot-to-foot [18] or on the cross-correlation [32] were used to estimate the transit time. Similar to the foot-to-foot approaches, the systolic up-slope was considered in our method rather than the entire curve as proposed in the cross-correlation methods. This choice was based on the unidirectional and reflectionless nature of the systolic up-slope [16,26] and aimed at minimizing the influence of the morphology of the downslope which may be altered by aortic stiffening and disorganization of the flow during end systole.

The reproducibility of the transit time estimation resulted in a low inter-operator variability as reflected by a coefficient of variation of 4.4%. In addition, the combination of this transit time with the estimated aortic arch length resulted in a reproducible estimate of *Arch_PWV. *Indeed the coefficient of variation obtained in the present study was lower than the coefficient of 13% presented in a previous study [18].

Furthermore, the estimated *Arch_PWV *was well correlated to the tonometric *CF_PWV*. However, values of *CF_PWV *were consistently higher. This can be explained by physiological reasons. Indeed, the elastic properties of the conduit arteries vary along the arterial tree, with more elastic proximal arteries and stiffer distal arteries [34-36]. Therefore PWV increases from central to peripheries arteries, for example from 4-5 m.s^-1 ^in the aortic arch to 8-9 m.s^-1 ^in the iliac and femoral arteries [37].

The transit time and subsequently the arch-PWV estimations could be influenced by the temporal resolution of the PC data. In the present study, breath-hold technique was used for the PC acquisition resulting in a lower temporal resolution compared to other validated free breathing acquisition techniques [18]. However, this later technique requires longer acquisition time and is exposed to more variation of the RR-interval during the acquisition as well as to the displacement of the aortic region of interest throughout the acquisition plane.

Another limitation is the use of *CF_PWV *values for comparison instead of invasive gold standard values. Indeed, it has been demonstrated that the *CF_PWV *calculation was influenced by the body shape and size [21]. However, it has been also demonstrated that in subjects with body mass index values <35 kg m^-2 ^the *CF_PWV *validly reflects aortic PWV values [38]. Therefore, because of the low body mass index of the studied population (23.75 ± 3.4 kg m^-2^), the effect of body shape was supposed to be minimal providing accurate *CF_PWV *values.

## Conclusions

Local and regional aortic stiffness indices were computed from a combination of CMR measurements with pulse pressure data. The consistency of these indices according to the theoretical model and to the well established tonometry measurement of the global aortic stiffness was demonstrated. The addition of such post processing techniques to the established CMR tools may prove clinically useful for the local evaluation of the aortic stiffness, especially in patients with localized aortic stiffness, such as subjects with Marfan disease. Therefore, CMR regional measurements in the ascending aorta and on the aortic arch should be clinically relevant because of the proximal position of these segments regarding to the left ventricle, and of their effects on its working conditions and consequently its physiopathology.

## Competing interests

The authors declare that they have no competing interests.

## Authors' contributions

AD participated in the conception and design of the study, in the technical developments, and in writing the manuscript. NK and FF participated in the conception and the revision of the manuscript. ML participated in data processing. ADC participated in the technical developments. EM participated in revising the manuscript, and in the acquisition and interpretation of data. AH participated in the technical developments, in the design and coordination of the study. All authors revised, read and approved the final version of the manuscript.
